# Migration Effects on Cognition: Protocol for the Aging in Kerala Americans Research Study

**DOI:** 10.2196/85493

**Published:** 2026-02-13

**Authors:** Kelly Cotton, Dristi Adhikari, Anne Felicia Ambrose, Emmeline Ayers, Helena M Blumen, Mirnova E Ceïde, VG Pradeep Kumar, Mairim Melecio-Vazquez, Sanish Sathyan, Alben Sigamani, Kavita Sivaramakrishnan, Marnina Stimmel, Erica F Weiss, Jie Yang, Joe Verghese

**Affiliations:** 1 Department of Neurology Renaissance School of Medicine Stony Brook University Stony Brook, NY United States; 2 Department of Neurology Albert Einstein College of Medicine Bronx, NY United States; 3 Department of Psychiatry and Behavioral Sciences Albert Einstein College of Medicine Bronx, NY United States; 4 Institute of Neurosciences Baby Memorial Hospital Kozhikode India; 5 Department of Molecular Biology Kannur University Kannur India; 6 Carmel Research Consultancy Pvt Ltd Bengaluru India; 7 Department of Sociomedical Sciences Mailman School of Public Health Columbia University New York, NY United States; 8 Department of Family, Population & Preventive Medicine Renaissance School of Medicine Stony Brook University Stony Brook, NY United States

**Keywords:** immigration, India, cognitive impairment, neuroimaging, aging, dementia, acculturation, biomarkers

## Abstract

**Background:**

In the United States, Asian American people represent the fastest growing population group, and are highly diverse linguistically, culturally, and demographically. Yet, in most national studies, Asian American groups are aggregated, masking potential health disparities. Racial and ethnic minorities, especially first-generation immigrants, are also at a particularly elevated risk of cognitive impairment.

**Objective:**

The Aging in Kerala Americans Research (AKKARE) study aims to examine both positive and negative migration effects on health in the first-generation Kerala American population, focusing on cognition and dementia. We will assess the effect of immigrant and cultural factors and social relations on cognitive aging from epidemiological, biological, and vascular perspectives. This protocol describes the study design and procedures for the AKKARE study.

**Methods:**

The AKKARE study proposes to enroll 400 older first-generation Kerala American individuals from the tristate area. A smaller subset of these participants will complete blood tests (n=360) and neuroimaging studies (n=160). We will assess the role of immigration and cultural effects on cognitive function, mood, and quality of life, as well as biological and vascular aging. We will conduct follow-up assessments at 12-month intervals for up to 5 years.

**Results:**

The AKKARE study (grant #1R01AG084567-01) was funded by the US National Institutes of Health in 2024 and received approval from the Stony Brook University Institutional Review Board to start the study in 2025. Enrollment began in September 2025.

**Conclusions:**

As there is presently a lack of fundamental data on the epidemiology in diseases of aging in Indian American immigrants, the AKKARE study will provide new insights into factors of risk and resilience associated with cognitive impairment in this group and in the broader older adult population.

**International Registered Report Identifier (IRRID):**

DERR1-10.2196/85493

## Introduction

### Background

In the United States, Asian American people represent the fastest-growing population group, more than doubling in size from 10.5 million in 2000 to more than 23 million in 2020. Asian American people trace their ancestry to at least 19 countries in East and Southeast Asia and the Indian subcontinent. The Asian American population (including Indian American people) is highly diverse linguistically, culturally, and demographically. In most national surveys and studies, however, these groups are aggregated, masking health disparities in specific groups.

The Indian American population is the second-largest immigrant group in the United States. The enactment of the 1965 Immigration and Naturalization law led to an uptick in immigration to the United States from India, and the Indian American population in the United States has increased dramatically, from 3000 in the 1960s to 4.6 million people in 2019 [1-3]. Immigrants from the state of Kerala in South India account for approximately 7% of the current Indian American population in the United States [1], particularly in the tristate area (New York, New Jersey, and Connecticut) [3]. Kerala has low per capita income (US $2775/year) and high unemployment (6%), but is on par with many developed countries in education (literacy rate 94%) and health [4,5], a paradox termed the “Kerala phenomenon” [4,6]. Our interviews and experience with Kerala American individuals reveal 2 major immigrant groups: one group who migrated to the United States in middle adulthood for education and job opportunities and have aged in place, and another group who migrated at older ages, following retirement in India, to join their children in the United States. Unlike the first group of migrants, the older second group has had less in-country time to establish social relations and assimilate the local culture. Hence, these 2 immigrant groups from Kerala likely vary in migrant experiences and social determinants of health.

The incidence of mild cognitive impairment and Alzheimer disease is high among racial or ethnic minorities, especially first-generation immigrants [7-10], and immigrant populations spend a greater proportion of their later years with cognitive impairment and dementia than native-born US populations [8,11]. Psychological stress, depression, and other migration-related factors (eg, age, acculturation, and social relations) may accelerate cognitive decline in migrants [7,12], but the situation is complex. Both worse health and better health outcomes (“healthy migrant effect”) are seen in migrants; however, benefits appear to wane with longer US residency [10,12-14]. Still, a 2021 trans–National Institutes of Health workshop on the Asian American population noted that there is a paucity of fundamental data on the epidemiology as well as risk and resilience factors in diseases of aging in this large US population segment.

**Figure 1:**
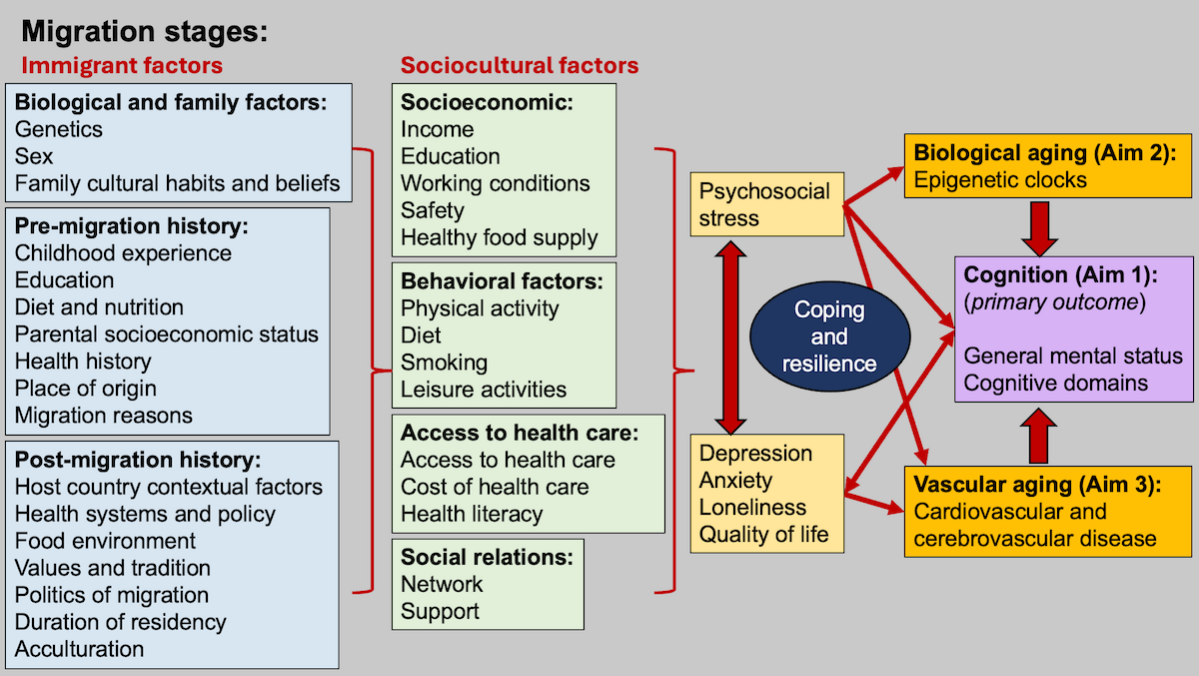
Conceptual model.

Here, we propose the Aging in Kerala Americans Research (AKKARE) study. In Malayalam, the language spoken by most people in Kerala, the word *akkare* translates to “on the other bank (shore),” and is also used to refer to overseas. In the AKKARE study, we aim to examine the effects, both positive and negative, of migrating to “the other bank” on health in first-generation Kerala American individuals, focusing on cognition and dementia. We will use a social-cultural conceptual model (Figure 1) to address migration effects on cognition that incorporates Zimmerman’s stages of migration (predeparture, travel, and destination phases; Table 1) [15] as well as social determinants of health. This conceptual visual model is meant to organize our study approach and to identify relationships between risk and protective factors for cognitive health in Kerala American individuals. We will focus on the effect of immigrant and cultural factors and social relations on cognitive aging from epidemiological, biological, and vascular perspectives.

**Table 1:** Measures and analyses by migration phase for each aim.

Migration phase	Predeparture	Destination	Predeparture	Destination	Predeparture	Destination
Study aim	Aim 1: identify immigrant and cultural factors and social relationships that contribute to dementia risk in older Kerala American people	Aim 1: identify immigrant and cultural factors and social relationships that contribute to dementia risk in older Kerala American people	Aim 2: determine the contribution of immigrant and cultural factors and social relationships to biological aging in older Kerala American people	Aim 2: determine the contribution of immigrant and cultural factors and social relationships to biological aging in older Kerala American people	Aim 3: determine the impact of immigrant and cultural factors and social relationships on vascular aging in older Kerala American people	Aim 3: determine the impact of immigrant and cultural factors and social relationships on vascular aging in older Kerala American people
Outcome	Global cognition, individual cognitive domains, mood, and quality of life	Global cognition, individual cognitive domains, mood, and quality of life	AgeAccel (biological aging)	AgeAccel (biological aging)	Blood pressure, ECG^a^, and small vessel disease	Blood pressure, ECG, and small vessel disease
Exposures	Age at migration, migration reasons, and marital status	Residency period in the United States, percent of life spent in the United States, occupation, acculturation, discrimination, stress, social network, perceived social support, and loneliness	Age at migration, migration reasons, and marital status	Residency period in the United States, percent of life spent in the United States, occupation, acculturation, discrimination, stress, social network, perceived social support, and loneliness	Age at migration, migration reasons, and marital status	Residency period in the United States, percent of life spent in the United States, occupation, acculturation, discrimination, stress, social network, perceived social support, and loneliness
Planned contrasts	Middle vs late-life migrants	Middle vs late-life migrants (United States vs Kerala cohorts)	Middle vs late-life migrants	Middle vs late-life migrants	Middle vs late-life migrants	Middle vs late-life migrants (United States vs Kerala cohorts)
Moderators	Diet, physical activity, leisure activity, smoking or alcohol use, and functional status	Diet, physical activity, leisure activity, smoking or alcohol use, and functional status	—^b^	—	—	—
Mediators	Biological aging and vascular markers	Biological aging and vascular markers	—	—	—	—
Covariates	—^c^	—^c^	—^c^	—^c^	—^c^	—^c^

^a^ECG: electrocardiogram.

^b^Not applicable.

^c^Shared across aims: age, sex, education, and chronic medical illness.

### Study Aims

There are 3 main aims of the AKKARE study. First, the study aims to identify immigrant and cultural factors and social relationships (such as social networks and social support) that contribute to the risk of dementia in older Kerala American individuals. We will assess the effects of immigrant and cultural factors (such as age at migration, residency duration, migration reasons, sex, education, and acculturation) on global cognition, as well as individual cognitive domains, mood, and quality of life. Furthermore, to address the “healthy migrant” effect [8], we will examine coping and resilience mechanisms that may help preserve cognition. The second aim is to determine the contribution of immigrant and cultural factors and social relationships to biological aging (quantified using biological clocks) in older Kerala American individuals. Third, the study aims to determine the impact of immigrant and cultural factors and social relationships on vascular aging in older Kerala American individuals. We will compare vascular aging (eg, cardiovascular and cerebrovascular indices) between the US and Kerala cohorts.

## Methods

### Study Design and Setting

We propose a research study of 400 older first-generation Kerala American individuals from the tristate area (New York, New Jersey, and Connecticut). A subset of participants will complete blood tests (n=360) and neuroimaging studies (n=160). We will also conduct follow-up assessments at 12-month intervals for up to 5 years.

### Eligibility Criteria

The general inclusion criteria include (1) age 60 years or older, (2) first-generation immigrant with at least 1 year US residency and currently living in the United States most (at least 6 months) of the year, (3) speaks Malayalam or English at a level sufficient to complete assessments, and (4) 1 participant per household to avoid bias on social measures.

The general exclusion criteria include (1) presence of dementia (Montreal Cognitive Assessment Blind/Telephone <10) [16]; inability to complete activities of daily living [17], or previous dementia diagnosis); (2) terminal illness with life expectancy <12 months, (3) progressive, degenerative, neurologic disease (eg, Parkinson disease and amyotrophic lateral sclerosis); and (4) major psychiatric symptoms, such as delusions or psychosis that will prevent assessments. Additionally, for participants who agree to participate in neuroimaging studies, the standard neuroimaging exclusion criteria are (1) presence of dementia; (2) presence of any surgically implanted metallic devices, such as aneurysm clips or pacemakers, and large amounts of dental or surgical hardware in the head and neck; and (3) a known history of claustrophobia.

### Recruitment

We will implement recruitment strategies with proven success in our center: outreach to Kerala American cultural and religious organizations, health fairs for the Kerala community, referrals from local clinicians, and targeted advertisements in Malayalam language print and online media. We will appoint a bilingual community outreach member to do outreach, recruitment, and engagement. The bilingual outreach staff member will engage with the community (eg, conducting needs assessment with participants, facilitating community engagement studios, and reaching out to community organizations), publicize the study, and promote enrollment.

### Assessment Procedures

Potential participants will be sent a letter describing the study, followed by a telephone call from bilingual research staff to assess interest and conduct a preliminary eligibility screen. The telephone interview includes verbal consent, confirmation of Kerala origin and US residency, and brief dementia screeners. We will arrange a home or clinic visit by our staff for potential recruits to obtain written informed consent and complete in-person assessments. We chose to offer the option of home-based or clinic-based testing for the convenience of participants, to build rapport, and to remove logistical barriers, such as travel, that may influence participation and retention. We will offer flexibility in the completion of self-report measures and questionnaires, and participants will complete these surveys on their own or remotely using technology (phone or internet), based on their preference. We will partner with a mobile phlebotomy and clinical exam company for participants to complete physical assessments, blood draw, and an electrocardiogram (ECG) on a separate day from the cognitive assessments. Transportation will be provided for a third visit to our research center for magnetic resonance imaging (MRI) studies. A subset of 15-20 participants, as well as community leaders, will also be invited to complete an additional day where participants will undergo interviews focused on ethnography and life course history. Participants will be compensated for their time.

### Measures

#### Sociodemographic

We will collect basic sociodemographic information (age, sex, education, income, and occupation) using a questionnaire. We will assess language use with the Language Experience and Proficiency Questionnaire [18], a valid and reliable tool for assessing language profiles of multilingual adults.

#### Cognition

We will assess cognition with validated tests in Malayalam and English, depending on participant preference. Cognitive testing will be administered by in-person bilingual research assistants. These tests overlap with the Kerala Einstein Study (KES), a cohort study of older adults residing in Kerala [19], facilitating cross-national comparisons.

Global cognition will be assessed with Addenbrooke’s Cognitive Examination (ACE)-III [20], a cognitive screening tool that incorporates the Mini-Mental State Examination as well as additional memory, language, visuospatial, and verbal fluency components, without specialized equipment, an advantage for home assessments. Scores on the ACE range from 0 to 100. The internal consistency, test-retest reliability, and interrater reliability of ACE were reported to be excellent worldwide [21,22]. The ACE predicts incident Alzheimer disease [23] and discriminates cognitively normal from mild cognitive impairment [20] and dementia [23,24]. The Malayalam version of the ACE has been previously validated [21,25].

We will also complete more robust assessments of individual cognitive domains, including memory, processing speed, executive function and attention, visuospatial reasoning, and verbal fluency (Table 2). Although participants will be able to choose their preference for language of interaction and assessment, we will evaluate and, if necessary, adjust for language proficiency in bilingual individuals by completing verbal fluency measures in both languages.

**Table 2:** Cognitive domains and associated tests.

Domain	Test
Global cognitive screener	Addenbrooke’s Cognitive Examination-III [20,21]
Memory	Verbal Learning Test^a^ [26,27] Picture Memory Impairment Screen [28,29] Modified Taylor Complex Figure, Recall Trials^a^ [26,30]
Processing speed	Symbol Match [31] Digit Symbol Substitution Test [32] Trail-Making (Black and White), Trial 1^a^ [26,33]
Executive function and attention	Digit Span [25,32] Trail-Making (Black and White), Trial 2^a^ [26,33]
Visuospatial reasoning	Modified Taylor Complex Figure copy^a^ [26,30]
Verbal fluency	Letter Fluency^a^ [26,34] Category Fluency^a^ [26]

^a^Test from the Indian Council of Medical Research-Neurocognitive Toolbox.

#### Mood

We will use the Geriatric Depression Scale (GDS)-15 [35] and Generalized Anxiety Disorder-7 item (GAD-7) [36] to assess symptoms of depression and anxiety, respectively. The Malayalam versions of the GDS and GAD-7 have been previously validated [37,38].

#### Quality of Life

Quality of life will be measured with the 12-Item Short-Form Health Survey, a well-validated and reliable scale [39]. The scale results in 2 summary scores—a mental component score and a physical component score.

#### Immigrant and Cultural Factors

Premigrant and postmigration factors will be collected. Premigration factors include sex, education, marital status, and migration reason (education, economic, or to join family). Postmigration factors include residency period in the United States, percent of life spent in the United States (years of residence in the United States divided by age), occupation, acculturation, discrimination, and stress. We will assess acculturation with the Mediators of Atherosclerosis in South Asians Living in America acculturation questionnaire, which was developed in South Asian immigrants [40]. The questionnaire asks participants, “How much would you wish these traditions from Kerala would be practiced in America?” with 7 items, including religious ceremonies, serving sweets at ceremonies, fasting on specific occasions, living in a joint family, arranged marriage, eating a traditional diet, and using spices for health and healing. Higher scores represent weaker traditional Indian beliefs. High Cronbach α (0.83) has been reported with similar reliability in men and women. Based on this scale, acculturation is coded into one of three categories: (1) assimilation (preference for US culture), (2) separation (preference for Kerala culture), and (3) integration (similar preference for both cultures).

We will measure perceived discrimination using the Everyday Discrimination Scale, a highly reliable scale [41]. The scale has 9 items that ask about mistreatment a participant may have experienced, such as “people act as if you are dishonest” and “you are threatened or harassed.” Participants respond to each item using a 6-point scale with options ranging from “never” (0) to “almost every day” (6).

Life stress will be measured using the Presumptive Stressful Life Events scale [42]. This scale includes 51 items about the occurrence of a variety of events in the past year and in their lifetime, such as having financial difficulties, family problems, job problems, or problems with people outside the family; suffering serious injury or the death of a spouse or friend; being a victim of a crime; and moving to a new home. Higher scores indicate greater life stress.

#### Social Relations

The Social Network Index (SNI) [43] will be used to quantify the number of high-contact social roles (SNI-1) a person interacts with at least biweekly and the total number of network members (SNI-2). A person can have a maximum of 12 high-contact social roles, including spouse, parent, child, child-in-law, close relative, close friend, religious group member, student, employee, neighbor, volunteer, and group member. The total number of network members is obtained by summing up the number of people a person interacts with at least biweekly across these 12 roles. Few high-contact social roles are defined as 4 or fewer social relationships that a person communicates with biweekly or more. A small network is defined as 18 or fewer social relationships.

We will assess perceived social support using the Medical Outcomes Study Social Support Survey [44], a validated and reliable social support scale that distinguishes between emotional or informational support, tangible support, affectionate support, and positive social interactions. The scale includes 19 items, such as “someone to share your most private worries and fears with” (emotional or informational), “someone to help you if you were confined to bed” (tangible), “someone who hugs you” (affectionate), and “someone to do something enjoyable with” (positive social interaction). For each item, participants are asked to indicate how often each type of support is available if needed, with options ranging from “None of the time” (1) to “All of the time” (5). Higher scores indicate higher levels of social support.

Finally, we will measure loneliness using the 3-item UCLA Loneliness scale [45,46]. This scale includes 3 items that assess 3 dimensions of loneliness: relational connectedness, social connectedness, and self-perceived isolation. For each item, participants indicate how often that item applies to them, (1) “Hardly ever,” (2) “Some of the time,” or (3) “Often.” Higher scores indicate greater loneliness.

#### Health and Lifestyle

We will collect information on medical history, medication use, and health care access. We will collect physical measurements (height, weight, and waist or hip circumference). We will assess health literacy using the Short Assessment of Health Literacy [47].

Traumatic brain injury history will be assessed using the Kerala Brain Injury Questionnaire, which is a reliable and valid assessment of chronic postconcussive symptoms in older Indian adults [48]. The Kerala Brain Injury Questionnaire can be used by nonclinicians, making it suitable for widespread case detection in community settings and for prescreening individuals before clinic visits, helping clinicians identify potential undiagnosed health issues.

We will assess function and disability with the Kerala Instrumental Activities of Daily Living scale [49,50], which has been shown to have excellent internal consistency and discriminative validity in Kerala [49,50].

We will assess cognitive and physical leisure activities using a previously validated scale [51,52]. We will collect information on current, past, and lifetime tobacco, marijuana, and alcohol use. We will assess dietary habits by asking participants to recall everything they ate or drank in the previous 24 hours, broken down by meal.

#### Physical Assessments

We will measure gait speed using a timed walk at a normal pace over a fixed distance (2.5 m). Use of any walking aid will be noted.

We will assess grip strength using a Jamar dynamometer. After identifying the dominant hand, the maximum voluntary contraction in the dominant hand will be measured.

#### Additional Questionnaires

We will assess subjective cognitive change using the Cognitive Change Index, which is a valid and consistent measure of self-perceived cognitive decline [53]. We will assess subjective change in memory with the 12 Cognitive Change Index items that assess memory performance (eg, “recalling information when I really try”). Participants are asked to compare their present ability to that of 5 years ago, rating each item from 1 (“no change/normal ability”) to 5 (“much worse/severe problem”). Higher scores indicate greater subjective cognitive decline. Participants will also complete a subjective cognitive and motor complaint questionnaire, which asks participants about mobility (eg, “How far can you walk in an hour?”) and cognitive ability (eg, “Daily problems with thinking and/or memory”).

#### Coping and Resilience Mechanisms

We will conduct semistructured topic-guide interviews that focus on life course histories and address topics such as acculturative stress, language history, and coping [54-56]. A bilingual interviewer will conduct hour-long home-based interviews with 15-20 Kerala American people, including participants, families, and community representatives or leaders, on days and timings of their convenience. Sampling with maximum diversity will be done, and people with different views will be selected. These interviews will be done on a separate day from the study visits. The interviews will be digitally recorded and transcribed verbatim.

#### Biological Aging

We will quantify biological aging using a DNA methylation (DNAm) clock. The clock is built from epigenetic DNAm markers that can accurately quantify an age-related phenotype or outcome, or both [57-61]. We will mainly use second-generation DNAm aging algorithms (GrimAge and PhenoAge), but we will adapt for all available epigenetic clocks, including first-generation clocks (Horvath [353CpGs] and Hannum Clocks [71CpGs] as well as any new clocks developed in the future with methylation data. DNAm clocks are built with a supervised machine learning method, such as a penalized regression trained against chronological age or on health-related outcomes to identify an informative and sparse predictive set of CpGs [61]. GrimAge was developed based on the 7 DNAm surrogates of plasma proteins (DNAm adrenomedullin levels, DNAm beta-2 microglobulin, DNAm cystatin C, DNAm growth differentiation factor 15, DNAm leptin, DNAm plasminogen activation inhibitor 1, and DNAm tissue inhibitor metalloproteinase 1) and smoking pack years in a 2-stage procedure. DNAm GrimAge is based on the 1030 CpGs methylation level. The GrimAge version 2 leverages 2 additional DNAm-based estimators of plasma proteins: high sensitivity log C-reactive protein and log hemoglobin A1C, and will also be tested in our participants. DNAm phenotypic age is another successful second-generation clock predicting aging outcomes, including all-cause mortality, health span, and Alzheimer disease. The epigenetic age calculation is carried out using the DNAm age website [62] or by using the R package (R Core Team), methylclock [63]. Epigenome-wide DNAm data are proposed to be generated using the Infinium Human MethylationEPIC v2.0 BeadChip (Illumina Inc). However, with ongoing advancements in methylation microarray technology, we plan to use the most updated array available at the time of profiling to ensure comprehensive and high-resolution data capture.

AgeAccel is defined as the residual resulting from regressing predicted age on chronological age, and is independent of chronological age [61,64]. A positive AgeAccel value indicates faster aging, and a negative value suggests slower aging. This measure is derived from clocks described above and will be used for our analyses.

#### Vascular Aging

We will assess vascular aging with 2 indices: cardiovascular (blood pressure and ECG) and cerebrovascular (small vessel disease). We will measure blood pressure as per the Systolic Blood Pressure Intervention Trial (SPRINT) protocol [65]. The participant will be positioned in a seated position with back support and arm at heart level. The trained mobile phlebotomists will measure blood pressure 3 times with an appropriately sized cuff after 3-5 minutes of quiet rest, and readings will be averaged. Blood pressure will be analyzed as a continuous value and using established high and low cut-scores.

We will use a 12-lead ECG at home to maximize data collection. This will be conducted on the same day as the blood draw. The 12-lead ECG systems are practical for home assessments as they are digital, compact, and portable. ECG traces will undergo technical review and be codified with Minnesota code classification as reported in KES [66]. ECG outcomes include left ventricular hypertrophy, abnormality of ST-segment and T-wave, and abnormal rhythms. All ECGs will be read and assessed for normality/abnormality by a trained physician. Participants will be informed of abnormal results.

The cerebrovascular index will focus on small vessel disease and includes lacunar infarctions, white matter hyperintensities, and microbleeds. Details of this measure are discussed further in this study.

### Neuroimaging: MRI Protocol and Assessment

We will assess cerebral small vessel disease with a focus on lacunar infarctions, white matter hyperintensities, and microbleeds. We will additionally measure cortical volume and thickness and white matter integrity to generate new hypotheses.

#### Image Acquisition

All MRI images will be acquired on a 3 Siemens PRISMA 3 Tesla Scanner (Siemens Healthineers). T1-weighted 3D-MP-RAGE images will be acquired over a 256 mm field of view (FOV) with echo time (TE)=2.49 milliseconds, repetition time (TR)=2260.0 milliseconds, 2.0 SENSE factor, and 1.0 mm isotropic resolution. Diffusion-weighted images will be acquired in 64 independent noncollinear directions over a 256 mm FOV with TE=69.0 milliseconds, TR=4200 milliseconds, and 2 mm slice thickness. 3D-FLAIR (fluid attenuating inverse recovery) will also be acquired over a 240 mm FOV with TE=326 milliseconds, TR=4300 milliseconds, and 1 mm slice thickness. 3D susceptibility-weighted images will be acquired with a TE=20.20 milliseconds, TR=27 milliseconds, 64 slices, and 0.86 voxel size. Finally, a resting-state functional MRI (or blood oxygenation level dependent) sequence will be acquired over a 224 mm FOV with TE=28.00 milliseconds, TR=2000 milliseconds, 2.0 mm slice thickness, and a multiband acceleration factor of 2.

#### Lacunar Infarctions

Lacunar infarctions are focal hyperintensities on T2-weighted or FLAIR images ≥3 mm in diameter. The whole brain will be visually searched for lacunar infarcts. Infarcts located in white matter must be hypointense on T1-weighted and FLAIR images to distinguish from white matter hyperintensities (discussed further in this study).

#### White Matter Hyperintensities

White matter hyperintensity (WMH) refers to the appearance on MRI of damage in white matter regions of the brain, and will be automatically quantified from 3D-FLAIR images using the lesion segmentation toolbox, which will be implemented with SPM12 (Wellcome Department of Imaging Neuroscience at University College London) or MATLAB (MathWorks) [67]. The lesion segmentation toolbox pipeline provides overall WMH lesion count and lesion volume, as well as a voxel-based WMH probability map that can be entered into subsequent analyses.

#### Cortical Microbleeds

Cortical microbleeds are small foci of chronic blood products in normal (or near normal) brain tissue. Microbleed Anatomical Rating Scale [68] will be applied to susceptibility-weighted images to rate definite and possible microbleeds in lobar, deep, and infratentorial regions [69].

#### Structural MRI

T1-weighted images will be parcellated into anatomical brain regions, which is important for examining cortical thickness and volume and a necessary step in processing data for examining structural connectivity (refer to “Diffusion-Weighted Imaging” section). Each T1-weighted image will be reconstructed using FreeSurfer (version 7.2; Martinos Center for Biomedical Imaging). FreeSurfer’s subcortical segmentation and cortical parcellation are comparable with manual labeling [70]. Each participant’s white and gray matter boundaries, as well as gray matter and cerebral spinal fluid boundaries, will be visually inspected slice by slice. The subcortical structure borders will be plotted with FreeView visualization tools (part of the FreeSurfer package), compared with actual brain regions, and manually corrected if necessary. Gray matter segmentation and longitudinal alignment will be performed with the FreeSurfer longitudinal pipeline, which is robust to initialization points and avoids biasing toward any 1 time point by generating a median template for each participant based on the T1-weighted images at both time points using cubic spline interpolation. Several processing steps, such as skull stripping, Talairach transforms, atlas registration, as well as spherical surface maps and parcellations, will then be initialized with common information from the subject-specific template, significantly increasing reliability and statistical power. Gray matter volumes and cortical thickness of 68 brain regions will be extracted from this FreeSurfer processing pipeline at each time point and then entered into subsequent statistical models. FreeSurfer 7.2 also provides additional measures that can be explored beyond the specific aims, including volumes of hippocampal and amygdalar subfields, subcortical and brain stem structures, and hypothalamic nuclei.

#### Diffusion-Weighted Imaging

Preprocessing will consist of field distortion corrections using top-up distributed as part of FSL (Functional Magnetic Resonance Imaging of the Brain Software Library) and eddy current and movement correction using the eddy tool in FSL. To evaluate any white matter changes, we will use TRACULA (Tracts Constrained by Underlying Anatomy) distributed as part of the FreeSurfer v.7.2 library [71,72], which uses probabilistic tractography to extract 42 major white matter tracts. The software performs informed automatic tractography by incorporating anatomical information from a training dataset, provided by the software, with the anatomical segmentation of the T1 image of the current dataset, thus increasing the accuracy of the white matter tract placement for each participant by incorporating each participant’s anatomical data into the tractography algorithm. Parcellation results from the FreeSurfer longitudinal stream will be applied to TRACULA to increase sensitivity to longitudinal changes in white matter tracts. The software outputs white matter integrity measures for each voxel inside the 42 tracts, with a mean of about 500 voxels per tract. Thus, for each participant, voxel-wise white matter integrity measures for 42 tracts at baseline and follow-up will be used in subsequent analyses.

### Blood Collection

The phlebotomy service will be provided with the names, addresses, and phone numbers of consenting participants and will be responsible for contacting them to schedule blood draw appointments. Collection materials will be mailed to participants’ homes in advance of the scheduled visit. Every effort will be made to conduct the blood draw within the same week or the following week of the interview. Fasting will be recommended and preferred, but not required. Phlebotomists will record the fasting status at the time of collection. The phlebotomy service will maintain regular communication with the study coordinator, reporting participants who decline to schedule appointments or miss scheduled visits. Follow-up efforts will be made to identify potential issues and to reschedule appointments when possible. Blood samples will be collected using both ethylenediaminetetraacetic acid (EDTA) tubes and serum separation tubes. EDTA tubes will be used for plasma isolation and for extracting DNA, which will be used for performing a methylation array for epigenetic aging clocks and other DNA-based analyses in the future. Phlebotomists will ship aliquots of plasma, serum, and EDTA whole blood overnight to the Stony Brook University *Freezer Farm*, a core facility that will ensure safe and reliable storage of samples at –80 °C for future research use. Serum and plasma will be stored for a wide range of biomarker analyses, including proteomic, metabolomic, and biochemical assays in the future. DNA will be extracted from EDTA whole blood using QIAamp DNA Blood Mini Kits (Qiagen), followed by quality assessment and quantification using the Agilent 2200 TapeStation or the Invitrogen Qubit fluorometer. Extracted DNA will also be stored at –80 °C until further analysis.

### Data Processing and Analysis

#### Quantitative Analyses

All analyses will be preceded by extensive data checking and verification to resolve any missing, inconsistent, or out-of-range values, followed by standard descriptive analyses to summarize variables. Transformation of continuous biomarkers will be considered if severe skewness in distributions is detected.

For aim 1, the primary analyses will evaluate cross-sectional associations of immigrant and cultural and social factors with global cognition (ACE-III score) and tests of episodic memory, executive function, and attention using a series of linear regression models and reported as adjusted beta estimates with 95% CIs. The predictors will be included as continuous scores or categorical variables. All models will be adjusted for age, sex, education, chronic medical illness, and other confounders. We will first examine predictors separately, as these relationships have not been established in the Kerala American population.

As a hypothesis-generating analysis for future directions, we will evaluate whether loneliness, diet, physical activity, and leisure activity moderate the association between immigration related predictors and cognition, by adding these variables and their interactions with the predictors in the linear regression models described above. We will perform mediation analysis using the product of coefficients method to test if any significant associations of immigrant, cultural, and social factors with cognition can be explained by a mediating effect of biological aging or vascular markers. We will also address sex in our analyses, both adjusting for it as a covariate and conducting stratified analysis by sex. Finally, we will conduct exploratory analyses to examine the association of predictors at baseline with longitudinal change on cognitive measures using linear mixed effects models adjusted for confounders. Given the sample size and limited follow-up, these analyses are exploratory and intended to generate new hypotheses.

To address the potential problems arising from multiple hypotheses being tested, we will examine correlations among the outcome variables and among the predictors, and use principal component analysis to reduce the dimensionality if the predictors are highly correlated. Then, we will prioritize the models being tested based on the literature and our data that is independent from this cohort. The relationship between variables will be visually examined, transformations on the variables will be performed, and extra terms will be added to the regression model if necessary to model nonlinear and nonmonotonic relationships.

Age acceleration is defined as the residual resulting from regressing predicted age on chronological age. For aim 2, the primary analysis will use linear regression models (adjusted for the same covariates described above) to examine the association of immigrant, cultural, or social measures with biological aging (age acceleration value). In addition, we will compare these predictors in those defined as fast agers and slow agers. A positive age acceleration value indicates faster aging, and a negative value suggests slower aging.

For aim 3, the primary analyses will use linear regression models to examine the association of immigrant and cultural factors and social relations with cardiovascular disease (blood pressure and ECG variables). Linear mixed effects models will be applied to cerebral small vessel disease (vascular and structural brain abnormality measures) to compare abnormalities between participants across strata of immigrant and cultural factors, as well as social relations, adjusting for confounders described above. As a hypothesis-generating analysis, we will perform a mediation analysis (product of coefficients method) to evaluate whether the vascular measures mediate any significant relationships seen in the primary analyses.

For all aims, we will contrast 2 Kerala American migrant groups (those who migrated in middle life and those who migrated in later life). For aims 1 and 3, we will also conduct a cross-national comparison of cognition and risk factors between the Kerala American and KES cohorts. We are unable to assess biological aging due to a lack of appropriate data in the KES cohort.

#### Qualitative Analyses

For the ethnographic life course history interviews, we will independently analyze textual data deductively and inductively and reach a consensus to minimize personal bias and interpretation errors, thereby ensuring validity and reliability. The study’s trustworthiness (ie, credibility, transferability, dependability, and confirmability) will be ensured by tracking the internal analysis processes. Data will be analyzed by the qualitative content analysis method with MaxQda software (2020; VERBI GmbH).

#### Power

Power estimation was performed for the primary hypothesis in each aim, which will all use baseline cross-sectional data and hence will not be affected by longitudinal attrition. For power calculation using multiple linear regressions, the detectable effect size was reported as the increase in *R*^2^, which is a standard and popularly used measure for effect size [73-75]. We did not translate such a change in *R*^2^ to the change or differences in any outcome because such numerical translation would require multiple, largely speculative assumptions about the unexplained variance and predictor distributions, and hence it could be misleading rather than clarifying, because it would suggest a level of precision about “clinically meaningful” changes that is not supported by available data. Power calculation considers confounders by assuming a *R*^2^ of 25%, the percentage of variance in the predictor of interest explained by confounders. With a sample size of 400 and estimated prevalence of predictors ranging from 15% to 40% based on KES and other Indian American cohort studies, we have greater than 80% power to detect an increase of *R*^2^=0.033 at a conservative significance level 0.005, chosen under the consideration of multiple testing. The optimal size for epigenetic studies (DNAm) to create an accurate calibration model for age estimation is estimated to be 134 individuals or more. Given the estimated sample of 360 Kerala American individuals with biological aging measures, we have >80% power to detect an increase of *R*^2^=0.032 at a conservative significance level of 0.01, with the consideration of multiple testing. We anticipate that all 400 participants will complete cardiovascular disease assessment (blood pressure and/or ECG). Given sample of 400, and at a conservative significance level of 0.005, we have >80% power to detect an increase of *R*^2^=0.033 for the linear model; and under the assumption of 20% prevalence of cardiovascular disease (either blood pressure or ECG abnormalities), 0.8 power to detect an odds ratio (OR) 1.66 with an increase of 1 SD of the predictor. In cognitively normal older adults in KES, the prevalence of hypertension was 31%, and ECG abnormalities varied from 4% to 16%, and higher prevalence was seen in dementia cases. Given the sample of 160 for MRIs, and under the same assumption, we have 0.8 power to detect an OR of 2.33 at a significance level of 0.005. We will also combine MRIs from KES and Kerala American cohorts to examine cerebrovascular disease. This combined 460 MRI samples (KES=300 and Kerala American=160), under the same assumptions, will provide >0.8 power to detect an OR 1.5 at a significance level of 0.005.

### Ethical Considerations

This study has been approved by the Stony Brook University Institutional Review Board (Stony Brook IRB 2025-00139). All participants will provide informed consent. Data will be deidentified, and protective measures will be taken to ensure participant privacy. Data will be maintained by assigning each new participant a unique study ID. That ID will be used in each database file to identify and link subject data. ID and name associations will be password-protected in an encrypted master file to which only the research staff have access. Participant data, including computer data disks, will be kept in a locked room. Identifying information about a subject will not be used during the discussion, presentation, or publication of any research data. Full backups of the database will be performed daily. For added security, copies of the database will be kept in 2 separate physical locations in locked, fire-resistant containers. Participants will be compensated for their time (US $25 per study visit, US $50 for blood collection visit, and US $50 for MRI visit).

## Results

The AKKARE study was funded by the US National Institutes of Health in 2024 and received approval from the Stony Brook University Institutional Review Board to start the study in 2025. We began recruitment in September 2025. As of January 2026, we enrolled 31 participants.

## Discussion

### Principal Findings

The proposed study aims to investigate the effects of migration on health in the Kerala American population, focusing on dementia risk and cognition. Studying Kerala American individuals can provide insights into risk and protection of cognition as well as Alzheimer and related dementia in other immigrant groups. Our interdisciplinary clinical and biological research will inform researchers and clinicians to better identify at-risk older US immigrants for targeted assessments and interventions, understand ethnic aging, introduce state-of-the-art biological investigations to immigrant health research, and create a cohort for future aging studies in the United States.

Various migration- and social-related factors can lead to physical as well as psychosocial stresses over months to years, and along with depression, apathy, anxiety, and loneliness, can result in adverse health and cognitive consequences, such as Alzheimer and related dementias. The term “resilience” generally refers to the capacity of individuals to survive in the face of stress and shocks [76], but it has not received much research attention in the context of cognition and dementia in migrants. In general, personal traits (self-esteem, motivation, optimism, intellect, and coping skills) and collective resources (community pride, ethnic networks, cultural practices, and faith-based networks) are recognized as protective factors that strengthen migrants’ capacity to overcome challenges [77-83]. These variables may buffer the negative impacts of stressful situations and life events on brain structure and networks [84,85]. Our study will use a mixed methods approach by implementing qualitative focus group interviews to provide context to our quantitative findings and to spur new research questions.

Aging is the biggest risk factor for dementia and is described in chronological or biological terms [86]. Chronological aging (number of years a person has been alive) increases at the same rate for everyone, whereas biological aging (how old a person seems) does not. However, chronological age by itself fails to explain the heterogeneity in health outcomes in individuals. For instance, individuals with the same chronological age can be either functionally independent or frail with multiple chronic diseases. Epigenetic aging is a novel measure of biological age that reflects exposures and disease risks independent of chronological age [57-59], and epigenetic clocks may be useful for early detection of adverse health outcomes in aging immigrants. Epigenetic modifications are theorized to help explain the persistence of racial and social health disparities, such as the increased burden of cardiovascular disease among Black American individuals relative to White American individuals [87]. Few studies have linked immigration status to age acceleration measures, as we propose to do. Epigenetic clocks are fairly new, and their relationship with cognitive aging and dementia is only beginning to be elucidated [64,88-91].

Cardiovascular disease (CVD) burden is increasing faster in developing countries, where it also occurs earlier in life [92-94]. Compared with migrants from other countries, Indian American people had the highest prevalence of overweight and were less physically active [95]. Further, chronic stress heightens risk for CVD [96]. Because racial or ethnic minorities face unique stressors (eg, acculturative stress and racial discrimination), the proposed study aims to test whether these experiences are linked to the presence of MRI markers of vascular pathology, and in the future, extend these findings to Alzheimer-related pathology. Disparities in Alzheimer disease and dementia are hypothesized to persist because immigrants have depleted resources to adapt to risks and brain pathology. Interventions aimed at vascular risks may prevent Alzheimer disease and dementia, especially in migrant and native US populations with high rates of CVD or unfavorable metabolic profiles.

The proposed study has several strengths. Most notably, our study is a first-of-its-kind study of cognition in first-generation Kerala American immigrants, and our previous KES study cohort will allow us to make cross-national comparisons. Furthermore, we will examine a breadth of factors related to immigration and culture, coping and resilience mechanisms, biological aging, and vascular aging to better understand the impact of migration on health. We also plan to create a biorepository for future biological studies. Finally, while our aims in this study are cross-sectional, we also propose longitudinal follow-up as a foundation for future studies of causality of Alzheimer disease and dementias, and this study provides the foundation for longitudinal epidemiological and biological studies of immigrant Asian American health in the United States.

### Conclusion

The AKKARE study will provide an in-depth understanding of migration effects on cognitive health in the Kerala American population, and shed light on underlying biological pathways and mechanisms, and interrelationships of biological and migrant related sociocultural factors. This research will have a major impact on our understanding of migrant health and Alzheimer risk among the fast-growing US population segment of Indian American individuals and lay the groundwork for future interventions to maintain cognitive health in other US immigrant populations.

## Appendix

Multimedia Appendix 1 Peer review report by AIMR - Aging, Injury, Musculoskeletal, and Rheumatologic Disorders Study Section, Population Sciences and Epidemiology Integrated Review Group (National Institutes of Health, USA).

## Abbreviations

ACE: Addenbrooke Cognitive Examination
AKKARE: Aging in Kerala Americans Research Study
CVD: cardiovascular disease
DNAm: DNA methylation
ECG: electrocardiogram
FLAIR: fluid-attenuated inversion recovery
FOV: field of view
FSL: Functional Magnetic Resonance Imaging of the Brain Software Library
GAD-7: Generalized Anxiety Disorder
GDS: Geriatric Depression Scale
KES: Kerala Einstein Study
MRI: magnetic resonance imaging
OR: odds ratio
SNI: Social Network Index
SPRINT: Systolic Blood Pressure Intervention Trial
TE: echo time
TR: repetition time
TRACULA: Tracts Constrained by Underlying Anatomy
WMH: white matter hyperintensity
