# Supplementary material for: Migration Effects on Cognition: Protocol for the Aging in Kerala Americans Research Study
Source: JMIR Res Protoc. 2026 Feb 13;15:e85493. doi: 10.2196/85493 (PMC12949402; doi:10.2196/85493)
Supplement: Multimedia Appendix 1 [file resprot_v15i1e85493_app1.pdf]

SUMMARY STATEMENT
(Privileged Communication)

PROGRAM CONTACT:
FRANK Bandiera
301-496-3131
frank.bandiera@nih.gov

Release Date: **08/09/2023**
Revised Date:

Application Number: **1R01AG084567-01**

Principal Investigator

VERGHESE, JOE

Applicant Organization: ALBERT EINSTEIN COLLEGE OF MEDICINE

Review Group: **AIMR**
Aging, Injury, Musculoskeletal, and Rheumatologic Disorders Study Section

Meeting Date: **07/12/2023**
Council: **OCT 2023**
Requested Start: **09/01/2023**

Opportunity Number: **PA-20-185**
PCC: **2CEPIFB**

Project Title: **Cognitive decline and dementia in older Kerala Americans**

SRG Action: **Impact Score:27 Percentile:13**
Next Steps: **Visit https://grants.nih.gov/grants/next_steps.htm**
Human Subjects: 30-Human subjects involved - Certified, no SRG concerns
Animal Subjects: 10-No live vertebrate animals involved for competing appl.
Gender: 1A-Both genders, scientifically acceptable
Minority: 1A-Minorities and non-minorities, scientifically acceptable
Age: 1A-Children, Adults, Older Adults, scientifically acceptable

Project Year	Direct Costs Requested	Estimated Total Cost
1	499,978	842,377
2	499,999	842,412
3	499,993	842,402
4	499,991	842,399
5	499,998	842,410
TOTAL	2,499,959	4,212,000

ADMINISTRATIVE BUDGET NOTE: The budget shown is the requested budget and has not been adjusted to reflect any recommendations made by reviewers. If an award is planned, the costs will be calculated by Institute grants management staff based on the recommendations outlined below in the COMMITTEE BUDGET RECOMMENDATIONS section.

1R01AG084567-01 VERGHESE, JOE

RESUME AND SUMMARY OF DISCUSSION: This proposal examines associations of migrant-related, socio-cultural factors, and cardiovascular disease with age-related cognitive decline in a cohort older native and American Kerala Indians. Reviewers considered this study significant because it will fill a knowledge gap of correlates of AD in an understudied population. Integration of socio-cultural factors with biological aging using epigenetic clocks was considered novel. Discussed strengths included use of a multi-national cohort, and measurement instruments validated in both languages. However, measurement of coping, resiliance, and cardiovascular aging were not considered robust. Well noted was the experienced research team with a track record in the study population. Reviewers weighted more the study's strengths and determined the study will likely have an overall high impact on filling our knowledge gap on correlates associated with age-related cognitive decline in migrant populations.

DESCRIPTION (provided by applicant): Asian Americans (including Indian Americans) comprise 6% of the U.S. population and represent the fastest growing population group. The incidence of mild cognitive impairment and Alzheimer's disease is higher among racial/ethnic minorities, especially in first-generation immigrants. Immigrant populations spend a greater proportion of their later years with cognitive impairment and dementia than native born U.S. populations. Yet, there are few epidemiological and biological studies of Alzheimer's disease and related dementias in immigrant populations such as Indian Americans. In this first of its kind study, we propose to clinically and biologically phenotype 400 older first-generation immigrants from the southern Indian state of Kerala residing in the tri-state area (New York, New Jersey, and Connecticut) to better understand risk and protection against Alzheimer's disease and related dementias in this fast-growing U.S. population segment. We also have an unique opportunity to compare Kerala American first-generation immigrants with a native Kerala based cohort of 800 older adults in our NIH funded Kerala-Einstein study. This cross-national study of a single racial/ethnic group from Kerala in native and immigrant settings using the same protocols will reduce variability and enhance discovery of Alzheimer's disease and dementia mechanisms. We hypothesize that immigration related sociocultural factors in older Kerala Americans will influence biological/vascular aging, which in turn will impact cognition. Aim 1: Determine role of immigrant/cultural factors and social relationships (networks and support) that contribute to Alzheimer's disease and dementia risk in older Kerala Americans. Immigrant/cultural factors include age at migration, residency duration, migration reasons, sex, education, acculturation, etc. Primary outcome is global cognition (Addenbrooke's Cognitive Examination: ACE). Secondary outcomes include individual cognitive domains, mood (depressive symptoms, anxiety) and quality of life. To address the 'healthy migrant' effect, we will examine coping and resilience mechanisms that may help preserve cognition. Aim 2: Determine the contribution of immigrant/cultural factors and social relationships to biological aging in older Kerala Americans. Biological aging is quantified using epigenetic clocks. Aim 3: Determine the impact of immigrant/cultural factors and social relationships on vascular aging in older Kerala Americans. Outcomes include cardiovascular (BP/ electrocardiogram) and cerebrovascular disease indices (MRI small vessel disease). We will compare U.S. and Kerala cohorts on vascular aging. Our proposal is very responsive to NOT-HL-23-001, which seek applications to understand health in Asian Americans. Studying Kerala Americans can provide insights into risk and protection of Alzheimer's disease and related dementias in other immigrant groups with high prevalence of cardiovascular disease.

PUBLIC HEALTH RELEVANCE: Asian Americans comprise 6% of the U.S. population and represent the fastest growing population group in U.S. The incidence of mild cognitive impairment and Alzheimer's Disease is higher among racial/ethnic minorities, especially in first generation immigrants. Yet, there are few epidemiological and biological studies of Alzheimer's disease and dementia in immigrant populations such as Indian Americans. In this first of its kind study, we propose to clinically and biologically phenotype 400 older first-generation immigrants from the southern Indian state of

Kerala residing in the tri-state area (New York, New Jersey, and Connecticut) to better understand risk and protection against cognitive decline and dementia in this fast-growing U.S. population segment.

CRITIQUE 1

Significance: 2
Investigator(s): 1
Innovation: 1
Approach: 3
Environment: 1

Overall Impact: This is a novel application by an experienced and accomplished investigator team which is designed to examine the role of immigrant/cultural factors and social relationships (social networks and supports) that contribute to risk of dementia in older Kerala (Asian Americans, Indian Americans). The study will also examine the contribution of immigrant status and cultural factors that contribute to dementia risk and examine the contribution of these to the epigenetic clock, a measure of biological aging, as well as vascular aging (measured by blood pressure, ECG, and MRI small vessel disease). The study will recruit 400 older fist-generation Kerala Americans in the tri-state area and also make comparisons with particpants in Kerala, leveraging existing work to establish a Kerala-based cohort of 800 participants. The work is highly novel and will be the first work to systematically examine cognition in first-generation Kerala Indian American immigrants, and is responsive to NOT-HL-23-001 for epidemiologic studies in Asian Americans. The translational nature of the proposed analyses utilizing the epigenetic clock also increase the novelty/innovation. This is an accomplished and experienced investigator team. The impact of this work if successful is deemed high.

1. Significance:

Strengths

- Examines the biological impact of immigration state and cultural factors and risk for cognitive impairment
- Although the study is focused on Kerala descent immigrants, the results would be informative about other immigrant populations

Weaknesses

- None noted by reviewer.

2. Investigator(s):

Strengths

- Accomplished, experienced investigator team
- Strong biostatistician on the team

Weaknesses

- None noted by reviewer.

3. Innovation:

Strengths

- First study to examine cognition in first-generation Kerala Americans

- Novel examination of epigenetic clock (biological aging) in relation to immigrant status and factors

Weaknesses

- None noted by reviewer.

4. Approach:

Strengths

- Study design including comparison of first-generation immigrants with Kerala-based participants
- Leveraging existing resource of 800 Kerala based participants

Weaknesses

- A limitation is that the epigenetic clock analyses are not feasible to be conducted in the Kerala-based population
- The proposed measures of cardiovascular disease/vascular aging are fairly simple. Understandable that these are for home collection (3-lead ECG), but for those who are able to attend a study visit in person, more in depth assessments of vascular aging would be feasible, i.e., vascular stiffness, echocardiogram
- Insufficient detail is provided about the qualitative interviews and how themes of resilience will be illicited

5. Environment:

Strengths

- Strong environment to support the proposed analyses

Weaknesses

- None noted by reviewer.

Study Timeline:

Strengths

- None noted by reviewer.

Weaknesses

- None noted by reviewer.

Protections for Human Subjects

Acceptable Risks and/or Adequate Protections

Data and Safety Monitoring Plan (Applicable for Clinical Trials Only):

Inclusion Plans

- Sex/Gender: Distribution justified scientifically
- Race/Ethnicity: Distribution not justified scientifically
- For NIH-Defined Phase III trials, Plans for valid design and analysis:

- Inclusion/Exclusion Based on Age: Distribution justified scientifically

Vertebrate Animals
Not Applicable (No Vertebrate Animals)

Biohazards
Not Applicable (No Biohazards)

Resubmission

Renewal

Revision

Applications from Foreign Organizations
Not Applicable (No Foreign Organizations)

Select Agents
Not Applicable (No Select Agents)

Resource Sharing Plans
Acceptable

Authentication of Key Biological and/or Chemical Resources
Not Applicable (No Relevant Resources)

Budget and Period of Support
Recommend as Requested

CRITIQUE 2

Significance: 2
Investigator(s): 1
Innovation: 2
Approach: 4
Environment: 1

Overall Impact: The PI and his team plan to study the role of immigrant/cultural factors and social relationships that may lead to cognitive impairment in older Kerala Americans. Several predictors such as age at immigration, education, their SES status, education will be assessed. Immigrant/cultural factors include age at migration, residency duration, migration reasons, sex, education, acculturation, etc. This aim will also look at decline in individual cognitive domains, mood, and quality of life as well as

resiliency. Aim 2 will determine contribution of cultural factors and social relationships to biological aging using epigenetic clocks. Aim 3 will study compare US and Kerala-Einstein cohorts to determine impact of immigration and related changes in lifestyle on vascular aging. The factors studied will include BP, ECG and MRI for cerebro-vascular and cardiovascular factors. The set of studies proposed are significant and meet NIH priority. Some weaknesses include largely observational aims, potential differences between ethnic groups which might affect generalizability. These weaknesses are relatively minor.

1. Significance:

Strengths

- Understand both positive and negative influences of migration on health
- Describe unique immigrant experiences in this population
- Understand social determinants of health (as affected by time spent in US) and its impact on this population particularly with respect to their cognitive health
- Study migration related factors and their influence on psychosocial and cognitive health
- Study influence of social connections in US and cultural connections in Kerala on resilience
- Good conceptual model
- Indian migrants have a significantly higher rate of cardiovascular disease and diabetes; this population is not well studied.
- Responsive to RFA

Weaknesses

- Conceptual model well thought out, but does test multiple associations and domains

2. Investigator(s):

Strengths

- PI is trained in neurology and geriatrics, has excellent track record on dementia, overall cognitive health and is well regarded in the field
- Long track record and expertise in this area of research
- PI has a prior NIH grant with a well-developed cohort of older adults in Kerala which will be used as a comparator cohort
- Supported with expertise in statistics, qualitative research, dementia
- Presence of infrastructure in Kerala with track record of collaboration

Weaknesses

- Variety of ethnic groups among SE Indians; diet, culture, social customs very different even within the same country – results from this may not necessarily generalizable to all from the subcontinent

3. Innovation:

Strengths

- Innovative questions to be asked with this population
- A unique population, not well-studied

- Use of epigenetic clock to study adverse influences of migration and other determinants of health
- Study innovative migrant experiences and its relation of vascular and cognitive health
- Reverse translation of measures developed in this population to a broader US population suggesting potential generalizability

Weaknesses

- None major
- Overall an observational study, but critically important to study and understand these factors before a larger interventional study

4. Approach:

Strengths

- Develop a cohort of 400 first generation Indian immigrants from Kerala in tri-state area
- Standard inclusion, exclusion criteria
- One visit to the participant’s home, second visit to the research center for blood work and MRI
- Availability of validated tools in Malayalum language; use of instruments developed using Kerala cohorts
- Predictors and approach to predictors are well-characterized and commensurate with the experience of the PI and team members.
- Exploration of immigration related psychosocial factors that can influence aging

Weaknesses

- Aims largely observational and although this population is less well-studied, would have benefitted from more innovative aims to study other aspects of engagement and recruitment as well as understanding other outcomes.
- Statistical analyses could be more in-depth considering the breadth and depth of this cohort and potential to ask several innovative questions

5. Environment:

Strengths

- No major concerns

Weaknesses

- None noted by reviewer.

Study Timeline:

Strengths

- None noted by reviewer.

Weaknesses

- None noted by reviewer.

Protections for Human Subjects

Acceptable Risks and/or Adequate Protections

- Adequate - protection during blood collection, MRI and other tests

Data and Safety Monitoring Plan (Applicable for Clinical Trials Only):
Not Applicable (No Clinical Trials)

Inclusion Plans

- Sex/Gender: Distribution justified scientifically
- Race/Ethnicity: Distribution justified scientifically
- For NIH-Defined Phase III trials, Plans for valid design and analysis: Not applicable
- Inclusion/Exclusion Based on Age: Distribution justified scientifically

Vertebrate Animals
Not Applicable (No Vertebrate Animals)

Biohazards
Not Applicable (No Biohazards)

Resubmission

Renewal

Revision

Applications from Foreign Organizations
Justified

Select Agents
Not Applicable (No Select Agents)

Resource Sharing Plans
Acceptable

Authentication of Key Biological and/or Chemical Resources
Not Applicable (No Relevant Resources)

Budget and Period of Support
Recommend as Requested

CRITIQUE 3

Significance: 1
Investigator(s): 1
Innovation: 2
Approach: 3
Environment: 1

Overall Impact: In this application, the investigators propose to study the factors associated with the incidence of mild cognitive impairment (MCI) and Alzheimer's Disease (AD) in first-generation immigrants to the USA hailing from Kerala, India. They have successfully run a study in Kerala over the past 15 years studying the same risks in Indian individuals living there. Specific aims address cultural factors and social environment (Aim 1), biological aging (Aim 2), and vascular aging (Aim 3) as putative factors contributing to both the risks of and protection from developing MCI and/or AD. Considerable emphasis is placed on data collection, analysis and interpretation that will allow future comparisons of health outcomes in immigrants from Kerala with the Indian population in Kerala itself. The study is significant due to a relatively small number of studies addressing the AD risks among racial/ethnic minorities. The study is innovative in its holistic view of changes to cognition among first-generation American immigrants who might be subject to the 'healthy migrant effect'. The investigative team is well qualified to carry out the proposed work on the scientific and practical fronts. Minor drawbacks include insufficient discussion of the approaches to address the bimodal distribution of the study participants (recent vs. established immigrants), multiple testing adjustments and relatively simplistic statistical models used to address the study aims. Overall, this project is considered to be of high impact.

1. Significance:

Strengths

- This study addresses the paucity of data collected and research undertaken on specific factors of risk and resilience to AD among Asian Americans
- Novel concepts of biological and vascular aging and their relationship with cognition are investigated

Weaknesses

- None noted by reviewer.

2. Investigator(s):

Strengths

- Dr. Verghese (PI) is a neurologist with expertise in a number of fields including geriatrics, neurophysiology, and study of dementia
- Extensive team of co-investigators has extensive complementary expertise in epidemiology and geriatric syndromes (Drs Ambrose, Ceide, and Ayers), neuroimaging (Dr. Blumen), neuropsychology (Dr. Weiss), biology of aging (Dr. Sathyan), social sciences (Drs Sivaramakrishnan and Blumen), biostatistics (Dr. Ye), and data management (Ms Ayers)

Weaknesses

- None noted by reviewer.

3. Innovation:

Strengths

- One of the few studies of cognition in Asian American immigrants

- Testing of the elements of a proposed comprehensive conceptual model of migration factors affecting cognition
- Study of the ‘healthy migrant effect’ as it relates to resilience to cognitive decline

Weaknesses

- None noted by reviewer.

4. Approach:

Strengths

- Principled and efficient cross-sectional and longitudinal study design built on a strong scientific premise
- Use of the validated instruments available both in Malayalam (majority language of Kerala) and in English
- Well-planned statistical analyses addressing the cross-sectional aspects of the study
- Use of novel epigenetic clocks to assess biological aging

Weaknesses

- Kerala Americans enrolled in the study come from two distinct immigrant groups arriving: (1) in middle adulthood and (2) at older ages. Sensitivity analysis addressing possible heterogeneity in responses is needed.
- Enrollment criteria on chronological age are the same in the US immigrants from Kerala and Kerala residents. However, the comparisons of biological age among the two groups are not addressed.
- Underdeveloped plans for addressing longitudinal aspects of the data addressing hypotheses in Aim 3
- Multiple testing adjustment is done in an ad-hoc way without truly accounting for the vast number of hypotheses tested
- Possible nonlinear associations cannot be discovered with a statistical methodology relying on linear regression which is pretty simplistic for the amount of data collected

5. Environment:

Strengths

- Research environment at the Albert Einstein College of Medicine is excellent to successfully achieve the study aims

Weaknesses

- None noted by reviewer.

Study Timeline:

Strengths

- None noted by reviewer.

Weaknesses

- None noted by reviewer.

Protections for Human Subjects

Acceptable Risks and/or Adequate Protections

Data and Safety Monitoring Plan (Applicable for Clinical Trials Only):

Not Applicable (No Clinical Trials)

Inclusion Plans

- Sex/Gender: Distribution justified scientifically
- Race/Ethnicity: Distribution justified scientifically
- For NIH-Defined Phase III trials, Plans for valid design and analysis: Not applicable
- Inclusion/Exclusion Based on Age: Distribution justified scientifically
- There is a justification provided to enroll exclusively Asian Americans

Vertebrate Animals

Not Applicable (No Vertebrate Animals)

Biohazards

Not Applicable (No Biohazards)

Resubmission

Renewal

Revision

Applications from Foreign Organizations

Not Applicable (No Foreign Organizations)

Select Agents

Not Applicable (No Select Agents)

Resource Sharing Plans

Acceptable

Authentication of Key Biological and/or Chemical Resources

Not Applicable (No Relevant Resources)

Budget and Period of Support

Recommend as Requested

THE FOLLOWING SECTIONS WERE PREPARED BY THE SCIENTIFIC REVIEW OFFICER TO SUMMARIZE THE OUTCOME OF DISCUSSIONS OF THE REVIEW COMMITTEE, OR REVIEWERS' WRITTEN CRITIQUES, ON THE FOLLOWING ISSUES:

PROTECTION OF HUMAN SUBJECTS: ACCEPTABLE
INCLUSION OF WOMEN PLAN: ACCEPTABLE

INCLUSION OF MINORITIES PLAN: ACCEPTABLE

INCLUSION ACROSS THE LIFESPAN: ACCEPTABLE

COMMITTEE BUDGET RECOMMENDATIONS: The budget was recommended as requested.

Footnotes for 1R01AG084567-01; PI Name: VERGHESE, JOE

NIH has modified its policy regarding the receipt of resubmissions (amended applications).See Guide Notice NOT-OD-18-197 at https://grants.nih.gov/grants/guide/notice-files/NOT-OD-18-197.html. The impact/priority score is calculated after discussion of an application by averaging the overall scores (1-9) given by all voting reviewers on the committee and multiplying by 10. The criterion scores are submitted prior to the meeting by the individual reviewers assigned to an application, and are not discussed specifically at the review meeting or calculated into the overall impact score. Some applications also receive a percentile ranking. For details on the review process, see http://grants.nih.gov/grants/peer_review_process.htm#scoring.

MEETING ROSTER

Aging, Injury, Musculoskeletal, and Rheumatologic Disorders Study Section
Population Sciences and Epidemiology Integrated Review Group
CENTER FOR SCIENTIFIC REVIEW
AIMR
07/12/2023 - 07/13/2023

Notice of NIH Policy to All Applicants: Meeting rosters are provided for information purposes only. Applicant investigators and institutional officials must not communicate directly with study section members about an application before or after the review. Failure to observe this policy will create a serious breach of integrity in the peer review process, and may lead to actions outlined in NOT-OD-22-044 at https://grants.nih.gov/grants/guide/notice-files/NOT-OD-22-044.html, including removal of the application from immediate review.

CHAIRPERSON(S)

MAESTRE, GLADYS E, PHD, MD
PROFESSOR
DEPARTMENTS OF NEUROSCIENCE AND HUMAN GENETICS
UNIVERSITY OF TEXAS RIO GRANDE VALLEY
BROWSNVILLE, TX 78520

MEMBERS

ALLEN, KELLI D, PHD
PROFESSOR
DEPARTMENT OF MEDICINE
UNIVERSITY OF NORTH CAROLINA AT CHAPEL HILL
CHAPEL HILL, NC 27599

AROKE, EDWIN NGOMUEH, PHD, MSN *
ASSOCIATE PROFESSOR
THE UNIVERSITY OF ALABAMA AT BIRMINGHAM
BIRMINGHAM, AL 35294

BANACK, HAILEY ROSE, PHD *
RESEARCH ASSISTANT PROFESSOR
DEPARTMENT OF EPIDEMIOLOGY
AND ENVIRONMENTAL HEALTH
BUFFALO, NY 14214

BERRY, SARAH D, MD, MPH
ASSCOCIATE PROFESSOR
DEPARTMENT OF MEDICINE
HEBREW REHABILITATION CENTER
HARVARD MEDICAL SCHOOL
BOSTON, MA 02131

BUSH, WILLIAM S, PHD
PROFESSOR
DEPARTMENT OF POPULATION AND
QUANTITATIVE HEALTH SCIENCES
CLEVELAND INSTITUTE FOR COMPUTATIONAL BIOLOGY
CASE WESTERN RESERVE UNIVERSITY
CLEVELAND, OH 44106

CHIRWA, SANIKA SAMUEL, PHD *
PROFESSOR
DEPARTMENT OF NEUROSCIENCE AND
PHARMACOLOGY
MEHARRY MEDICAL COLLEGE
VANDERBILT UNIVERSITY
NASHVILLE, TN 37208

DONNEYONG, MACARIUS M, PHD, MPH *
ASSOCIATE PROFESSOR
DIVISION OF OUTCOMES AND TRANSLATIONAL SCIENCES
COLLEGE OF PHARMACY
THE OHIO STATE UNIVERSITY
COLUMBUS, OH 43026

ELLIS, RONALD J, PHD
PROFESSOR
DEPARTMENT OF NEUROSCIENCES
SCHOOL OF MEDICINE
UNIVERSITY OF CALIFORNIA, SAN DIEGO
SAN DIEGO, CA 92103

ESPINOZA, SARA ELYSE, MD *
PROFESSOR
DEPARTMENT OF MEDICINE
CEDARS-SINAI MEDICAL CENTER
LOS ANGELES, CA 90048

GAJEWSKI, BYRON J, PHD
PROFESSOR
DEPARTMENT OF BIOSTATISTICS AND DATA SCIENCE
SCHOOL OF MEDICINE
UNIVERSITY OF KANSAS
KANSAS CITY, KS 66160

GOODE, ADAM, PHD, DPT *
ASSOCIATE PROFESSOR
SCHOOL OF MEDICINE
DUKE UNIVERSITY
DURHAM, NC 27708

HALL, CHARLES B, PHD, MS *
PROFESSOR
DIVISION OF BIOSTATISTICS
DEPT OF EPIDEMIOLOGY AND POPULATION HEALTH
SAUL R. KOREY DEPARTMENT OF NEUROLOGY
ALBERT EINSTEIN COLLEGE OF MEDICINE
BRONX, NY 10461

HAREZLAK, JAROSLAW, PHD
PROFESSOR
DEPARTMENT OF EPIDEMIOLOGY AND BIOSTATISTICS
SCHOOL OF PUBLIC HEALTH-BLOOMINGTON
INDIANA UNIVERSITY
BLOOMINGTON, IN 47405

HAUSDORFF, JEFFREY M, PHD, MS *
PROFESSOR
FACULTY OF MEDICINE, TEL AVIV UNIVERSITY
NEUROLOGICAL INSTITUTE, TEL AVIV MEDICAL CENTER
RADC AND DEPARTMENT OF ORTHOPAEDIC SURGERY
RUSH UNIVERSITY MEDICAL CENTER
TEL AVIV 64239
ISRAEL

HICKS, GREGORY E, PHD, MPT *
DISTINGUISHED PROFESSOR OF HEALTH SCIENCES
DEPARTMENT OF PHYSICAL THERAPY
UNIVERSITY OF DELAWARE
NEWARK, DE 19713

HUELS, ANKE, PHD, MS *
ASSISTANT PROFESSOR
DEPARTMENT OF EPIDEMIOLOGY AND GANGAROSA
DEPARTMENT OF ENVIRONMENTAL HEALTH
ROLLINS SCHOOL OF PUBLIC HEALTH, EMORY UNIVERSITY
ATLANTA, GA 30322

KAWAI, VIVIAN K, MD *
ASSISTANT PROFESSOR
DEPARTMENT OF MEDICINE
DIVISION OF CLINICAL PHARMACOLOGY
VANDERBILT UNIVERSITY MEDICAL CENTER
NASHVILLE, TN 37203

MCGRATH, RYAN, PHD *
ASSISTANT PROFESSOR
DEPARTMENT OF HEALTH, NUTRITION, AND
EXERCISE SCIENCES
NORTH DAKOTA STATE UNIVERSITY
FARGO, ND 58108

MODY, LONA, MD
PROFESSOR
DEPARTMENT OF EPIDEMIOLOGY
SCHOOL OF PUBLIC HEALTH
UNIVERSITY OF MICHIGAN AT ANN ARBOR
ANN ARBOR, MI 48109

ODDEN, MICHELLE C, PHD
ASSOCIATE PROFESSOR
DEPARTMENT OF EPIDEMIOLOGY AND POPULATION
HEALTH
STANFORD UNIVERSITY
STANFORD, CA 94305

PETROVSKY, DARINA V, PHD *
ASSISTANT PROFESSOR
DIVISION OF NURSING SCIENCE
RUTGERS UNIVERSITY
PHILADELPHIA, PA 19104

RAJI, CYRUS A, PHD, MD *
ASSISTANT PROFESSOR
DEPARTMENT OF RADIOLOGY AND NEUROLOGY
WASHINGTON UNIVERSITY IN ST. LOUIS
SAINT LOUIS, MO 63108

RIANON, NAHID J, PHD *
PROFESSOR AND MEMORIAL HERMANN ENDOWED CHAIR
DEPARTMENT OF FAMILY & COMMUNITY MEDICINE
JOAN AND STANFORD ALEXANDER DIVISION OF GERIATRIC
PALLIATIVE MEDICINE/INTERNAL MEDICINE
THE UNIVERSITY OF TEXAS HEALTH SCIENCE CENTER AT H
HOUSTON, TX 77030

SARKIS, RANI, MD, MS *
ASSISTANT PROFESSOR OF NEUROLOGY
DIVISION OF EPILEPSY
DEPARTMENT OF NEUROLOGY
BRIGHAM AND WOMEN'S HOSPITAL
HARVARD MEDICAL SCHOOL
BOSTON, MA 02115

SINGH, JASVINDER A, MPH, MBBS *
STAFF RHEUMATOLOGIST, BIRMINGHAM VETERANS
AFFAIRS MEDICAL CENTER
ENDOWED PROFESSOR
MUSCULOSKELETAL OUTCOMES RESEARCH
DIVISION OF CLINICAL IMMUNOLOGY AND RHEUMATOLOGY
UNIVERSITY OF ALABAMA AT BIRMINGHAM
BIRMINGHAM, AL 35233

VARDARAJAN, BADRI N, PHD, MS *
ASSISTANT PROFESSOR
DEPARTMENT OF NEUROLOGY
GERTRUDE H. SERGIEVSKY CENTER
COLUMBIA UNIVERSITY
NEW YORK, NY 10032

ZHAO, JINYING, PHD, MD *
DEANS ENDOWED CHAIR AND PROFESSOR
DIRECTOR, CENTER FOR GENETIC EPIDEMIOLOGY
& BIOINFOMATICS
COLLEGE OF PUBLIC HEALTH & HEALTH PROFESSIONS
UNIVERSITY OF FLORIDA
GAINESVILLE, FL 32610

SCIENTIFIC REVIEW OFFICER

FORBANG, NKETI I, MD, MPH
SCIENTIFIC REVIEW OFFICER
CENTER FOR SCIENTIFIC REVIEW
NATIONAL INSTITUTES OF HEALTH
BETHESDA, MD 20892

EXTRAMURAL SUPPORT ASSISTANT

BALOGUN, OLIVIA YVONNE DAMILOLA ADESEWA
EXTRAMURAL SUPPORT ASSISTANT
CENTER FOR SCIENTIFIC REVIEW
NATIONAL INSTITUTES OF HEALTH
BETHESDA, MD 20892

* Temporary Member. For grant applications, temporary members may participate in the entire meeting or may review only selected applications as needed.

Consultants are required to absent themselves from the room during the review of any application if their presence would constitute or appear to constitute a conflict of interest.
